# Poly[diaqua­(μ_2_-oxalato-κ^4^ *O* ^1^,*O* ^2^:*O* ^1′^,*O* ^2′^)(μ_2_-pyrazine-2-carboxyl­ato-κ^4^ *N* ^1^,*O*:*O*,*O*′)neodymium(III)]

**DOI:** 10.1107/S1600536809031250

**Published:** 2009-08-12

**Authors:** Ke-Chun Chen, Huan-Mian Luo, Qiu-Hui Meng, Yi-Fan Luo, Rong-Hua Zeng

**Affiliations:** a School of Chemistry and Environment, South China Normal University, Guangzhou 510006, People’s Republic of China; b Key Laboratory of the Technology of Electrochemical Energy Storage and Power Generation in Guangdong Universities, South China Normal University, Guangzhou 510006, People’s Republic of China

## Abstract

In the title complex, [Nd(C_5_H_3_N_2_O_2_)(C_2_O_4_)(H_2_O)_2_]_*n*_, the Nd^III^ atom is ten-coordinated by one N atom and three O atoms from two pyrazine-2-carboxyl­ate ligands, four O atoms from two oxalate ligands and two water mol­ecules in a distorted bicapped square-anti­prismatic geometry. The two crystallographically independent oxalate ligands, each lying on an inversion center, act as bridging ligands, linking Nd atoms into an extended zigzag chain. Neighboring chains are linked by the pyrazine-2-carboxyl­ate ligands into a two-dimensional layerlike network in the (10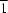) plane. The layers are further connected by O—H⋯O and O—H⋯N hydrogen bonds, forming a three-dimensional supra­molecular network.

## Related literature

For general background to lanthanide coordination frameworks, see: Han *et al.* (2009 ▶); Li *et al.* (2006 ▶); Wang *et al.* (2006 ▶); Zhou *et al.* (2006 ▶).
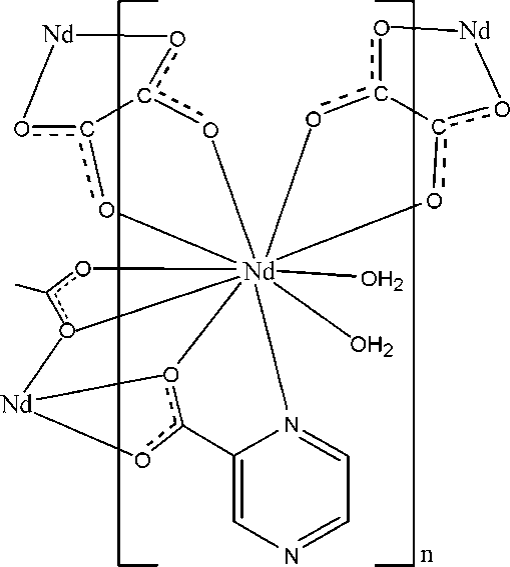


## Experimental

### 

#### Crystal data

[Nd(C_5_H_3_N_2_O_2_)(C_2_O_4_)(H_2_O)_2_]
*M* *_r_* = 391.39
Triclinic, 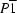

*a* = 7.948 (3) Å
*b* = 8.6512 (18) Å
*c* = 8.7425 (18) Å
α = 115.525 (2)°
β = 101.970 (3)°
γ = 96.306 (3)°
*V* = 517.0 (2) Å^3^
*Z* = 2
Mo *K*α radiation
μ = 5.06 mm^−1^
*T* = 296 K
0.23 × 0.19 × 0.17 mm

#### Data collection

Bruker APEXII CCD diffractometer
Absorption correction: multi-scan (*SADABS*; Sheldrick, 1996 ▶) *T*_min_ = 0.320, *T*_max_ = 0.420
2533 measured reflections
1824 independent reflections
1756 reflections with *I* > 2σ(*I*)
*R*_int_ = 0.065

#### Refinement

*R*[*F* ^2^ > 2σ(*F* ^2^)] = 0.051
*wR*(*F* ^2^) = 0.132
*S* = 1.08
1824 reflections
175 parameters
6 restraints
H atoms treated by a mixture of independent and constrained refinement
Δρ_max_ = 1.95 e Å^−3^
Δρ_min_ = −2.98 e Å^−3^

### 

Data collection: *APEX2* (Bruker, 2007 ▶); cell refinement: *SAINT* (Bruker, 2007 ▶); data reduction: *SAINT*; program(s) used to solve structure: *SHELXS97* (Sheldrick, 2008 ▶); program(s) used to refine structure: *SHELXL97* (Sheldrick, 2008 ▶); molecular graphics: *DIAMOND* (Brandenburg, 1999 ▶); software used to prepare material for publication: *SHELXTL* (Sheldrick, 2008 ▶).

## Supplementary Material

Crystal structure: contains datablocks I, global. DOI: 10.1107/S1600536809031250/hy2217sup1.cif

Structure factors: contains datablocks I. DOI: 10.1107/S1600536809031250/hy2217Isup2.hkl

Additional supplementary materials:  crystallographic information; 3D view; checkCIF report

## Figures and Tables

**Table 1:** Selected bond lengths (Å)

Nd1—O2*W*	2.467 (5)
Nd1—O3	2.474 (5)
Nd1—O6^i^	2.490 (5)
Nd1—O4^ii^	2.508 (5)
Nd1—O5	2.512 (5)
Nd1—O1*W*	2.549 (6)
Nd1—O2^iii^	2.557 (5)
Nd1—O2	2.573 (5)
Nd1—N1^iii^	2.765 (6)
Nd1—O1	2.885 (6)

Symmetry codes: (i) 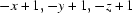; (ii) 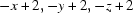; (iii) 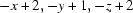.

**Table 2:** Hydrogen-bond geometry (Å, °)

*D*—H⋯*A*	*D*—H	H⋯*A*	*D*⋯*A*	*D*—H⋯*A*
O1*W*—H1*W*⋯O1^iv^	0.84 (9)	2.02 (6)	2.707 (8)	139 (7)
O1*W*—H2*W*⋯O6^v^	0.85 (8)	2.07 (4)	2.838 (8)	152 (6)
O2*W*—H3*W*⋯N2^vi^	0.84 (5)	2.50 (5)	3.229 (9)	145 (6)
O2*W*—H4*W*⋯O4^vii^	0.84 (8)	2.13 (4)	2.872 (8)	147 (7)

Symmetry codes: (iv) 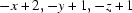; (v) 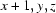; (vi) 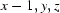; (vii) 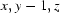.

**Fig. 1.**
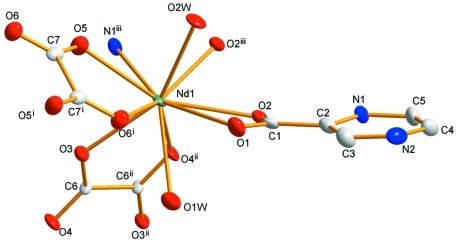
The asymmetric unit of the title compound. Displacement ellipsoids are drawn at the 30% probability level. H atoms have been omitted for clarity. [Symmetry codes: (i) 1-x, 1-y, 1-z; (ii) 2-x, 2-y, 2-z; (iii) 2-x, 1-y, 2-z.]

**Fig. 2.**
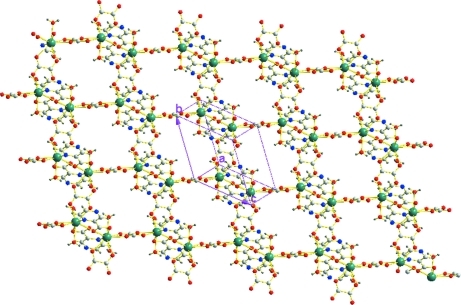
View of the layered network in the title compound.

## supplementary crystallographic information

### Comment

In recent years, many research groups have devoted their work to the design and synthesis of lanthanide coordination frameworks with bridging multifunctional organic ligands, not only because of their fascinating topological networks, but also due to their potential applications in ion exchange, gas storage, catalysis and luminescence (Wang *et al.*, 2006; Zhou *et al.*, 2006). As a building block, pyrazine-2-carboxylic acid and oxalic acid are good ligands with multifunctional coordination sites providing a high likelihood for the generation of structures with high dimensions (Han *et al.*, 2009; Li *et al.*, 2006). Recently, we obtained the title coordination polymer under hydrothermal conditions.

In the title compound, the Nd^III^ atom is coordinated by seven O atoms and one N atom from two pyrazine-2-carboxylate ligands and two oxalate ligands, and by two water molecules in a distorted bicapped square-antiprismatic geometry (Fig. 1). The Nd^III^ atoms are linked by the oxalate ligands, forming an extended zigzag chain. These chains are linked by the pyrazine-2-carboxylate ligands into a two-dimensional layerlike network (Fig. 2). The Nd—O and Nd—N bond lengths range from 2.467 (4) to 2.885 (6) Å (Table 1). O—H···O and O—H···N hydrogen bonds (Table 2) involving the pyrazine-2-carboxylate ligands, coordinated water molecules and oxalate ligands assemble neighboring layers into a three-dimensional supramolecular network.

### Experimental

A mixture of Nd_2_O_3_ (0.245 g, 0.75 mmol), pyrazine-2-carboxylic acid (0.186 g, 1.5 mmol), oxalic acid (0.135 g, 1.5 mmol), water (10 ml) in the presence of HNO_3_ (0.024 g, 0.385 mmol) was stirred vigorously for 20 min and then sealed in a Teflon-lined stainless-steel autoclave (20 ml capacity). The autoclave was heated and maintained at 433 K for 3 d, and then cooled to room temperature at 5 K h^-1^. Colorless block crystals of the title compound were obtained.

### Refinement

Water H atoms were tentatively located in difference Fourier maps and were refined with distance restraints of O—H = 0.84 (1) and H···H = 1.34 (1) Å , and with a fixed *U*_iso_(H). C-bound H atoms were placed at calculated positions and were treated as riding on the parent C atoms, with C—H = 0.93 Å, and with *U*_iso_(H) = 1.2*U*_eq_(C). The highest residual electron density was found 0.96 Å from Nd1 and the deepest hole 0.87 Å from Nd1.

### Crystal data

**Table:**

[Nd(C_5_H_3_N_2_O_2_)(C_2_O_4_)(H_2_O)_2_]	*Z* = 2
*M**_r_* = 391.39	*F*(000) = 374
Triclinic, *P*1	*D*_x_ = 2.514 Mg m^−^^3^
Hall symbol: -P 1	Mo *K*α radiation, λ = 0.71073 Å
*a* = 7.948 (3) Å	Cell parameters from 2522 reflections
*b* = 8.6512 (18) Å	θ = 2.7–28.3°
*c* = 8.7425 (18) Å	µ = 5.06 mm^−^^1^
α = 115.525 (2)°	*T* = 296 K
β = 101.970 (3)°	Block, colorless
γ = 96.306 (3)°	0.23 × 0.19 × 0.17 mm
*V* = 517.0 (2) Å^3^	

### Data collection

**Table:**

Bruker APEXII CCD diffractometer	1824 independent reflections
Radiation source: fine-focus sealed tube	1756 reflections with *I* > 2σ(*I*)
graphite	*R*_int_ = 0.065
φ and ω scans	θ_max_ = 25.2°, θ_min_ = 2.7°
Absorption correction: multi-scan (*SADABS*; Sheldrick, 1996)	*h* = −8→9
*T*_min_ = 0.320, *T*_max_ = 0.420	*k* = −8→10
2533 measured reflections	*l* = −10→10

### Refinement

**Table:**

Refinement on *F*^2^	Primary atom site location: structure-invariant direct methods
Least-squares matrix: full	Secondary atom site location: difference Fourier map
*R*[*F*^2^ > 2σ(*F*^2^)] = 0.051	Hydrogen site location: inferred from neighbouring sites
*wR*(*F*^2^) = 0.132	H atoms treated by a mixture of independent and constrained refinement
*S* = 1.08	*w* = 1/[σ^2^(*F*_o_^2^) + (0.0478*P*)^2^ + 0.0313*P*] where *P* = (*F*_o_^2^ + 2*F*_c_^2^)/3
1824 reflections	(Δ/σ)_max_ = 0.002
175 parameters	Δρ_max_ = 1.95 e Å^−^^3^
6 restraints	Δρ_min_ = −2.98 e Å^−^^3^

### Fractional atomic coordinates and isotropic or equivalent isotropic displacement parameters (Å^2^)

**Table:**

	*x*	*y*	*z*	*U*_iso_*/*U*_eq_	
C1	1.1008 (9)	0.3421 (10)	0.7510 (9)	0.0146 (15)	
C2	1.2220 (10)	0.2213 (10)	0.7476 (10)	0.0175 (15)	
C3	1.2449 (13)	0.0959 (12)	0.5928 (12)	0.0246 (19)	
H3	1.1882	0.0920	0.4865	0.030*	
C4	1.4345 (11)	0.0053 (11)	0.7489 (11)	0.0227 (17)	
H4	1.5126	−0.0660	0.7543	0.027*	
C5	1.4166 (11)	0.1312 (11)	0.9049 (11)	0.0235 (17)	
H5	1.4818	0.1415	1.0116	0.028*	
C6	0.9283 (8)	1.0062 (9)	0.9289 (9)	0.0114 (14)	
C7	0.4413 (9)	0.5252 (9)	0.5645 (9)	0.0156 (15)	
H1W	1.058 (11)	0.745 (10)	0.649 (9)	0.023*	
H2W	1.143 (10)	0.630 (10)	0.676 (10)	0.023*	
H3W	0.599 (5)	0.229 (10)	0.680 (9)	0.023*	
H4W	0.731 (9)	0.226 (9)	0.802 (10)	0.023*	
N1	1.3077 (9)	0.2389 (9)	0.9065 (8)	0.0175 (13)	
N2	1.3439 (9)	−0.0191 (9)	0.5892 (9)	0.0240 (15)	
Nd1	0.83941 (4)	0.58653 (4)	0.82456 (4)	0.0124 (2)	
O1	1.0011 (8)	0.3228 (8)	0.6106 (7)	0.0244 (13)	
O2	1.1050 (7)	0.4616 (7)	0.9021 (7)	0.0182 (11)	
O3	0.8094 (7)	0.8693 (7)	0.8272 (7)	0.0185 (11)	
O4	0.9419 (7)	1.1485 (7)	0.9251 (7)	0.0192 (11)	
O5	0.5149 (7)	0.5678 (8)	0.7214 (7)	0.0230 (12)	
O6	0.2843 (7)	0.5193 (8)	0.4984 (6)	0.0210 (11)	
O1W	1.0754 (9)	0.6971 (9)	0.7148 (8)	0.0240 (13)	
O2W	0.7008 (7)	0.2834 (7)	0.7480 (7)	0.0204 (11)	

### Atomic displacement parameters (Å^2^)

**Table:**

	*U*^11^	*U*^22^	*U*^33^	*U*^12^	*U*^13^	*U*^23^
C1	0.019 (4)	0.013 (4)	0.016 (4)	0.002 (3)	0.009 (3)	0.009 (3)
C2	0.024 (4)	0.013 (4)	0.016 (4)	0.001 (3)	0.005 (3)	0.007 (3)
C3	0.034 (5)	0.016 (5)	0.020 (4)	0.005 (4)	0.007 (4)	0.006 (4)
C4	0.028 (4)	0.016 (4)	0.028 (4)	0.010 (3)	0.016 (4)	0.010 (4)
C5	0.025 (4)	0.024 (4)	0.030 (4)	0.011 (3)	0.009 (3)	0.018 (4)
C6	0.008 (3)	0.006 (3)	0.014 (3)	−0.001 (2)	−0.001 (3)	0.001 (3)
C7	0.018 (4)	0.006 (4)	0.016 (4)	0.000 (3)	0.007 (3)	−0.001 (3)
N1	0.023 (3)	0.015 (3)	0.018 (3)	0.008 (3)	0.007 (3)	0.009 (3)
N2	0.029 (4)	0.013 (3)	0.027 (4)	0.003 (3)	0.014 (3)	0.004 (3)
Nd1	0.0159 (3)	0.0065 (3)	0.0146 (3)	0.00016 (19)	0.0035 (2)	0.0058 (2)
O1	0.032 (3)	0.027 (3)	0.021 (3)	0.010 (3)	0.009 (2)	0.016 (3)
O2	0.022 (3)	0.014 (3)	0.024 (3)	0.006 (2)	0.011 (2)	0.012 (2)
O3	0.016 (3)	0.013 (3)	0.024 (3)	0.000 (2)	0.000 (2)	0.010 (2)
O4	0.025 (3)	0.010 (3)	0.024 (3)	0.002 (2)	0.003 (2)	0.011 (2)
O5	0.022 (3)	0.033 (3)	0.019 (3)	0.009 (2)	0.008 (2)	0.014 (3)
O6	0.019 (3)	0.024 (3)	0.015 (3)	0.004 (2)	0.003 (2)	0.006 (2)
O1W	0.033 (3)	0.022 (3)	0.022 (3)	0.008 (3)	0.013 (3)	0.012 (3)
O2W	0.029 (3)	0.007 (3)	0.022 (3)	−0.003 (2)	0.006 (2)	0.006 (2)

### Geometric parameters (Å, °)

**Table:**

C1—O1	1.246 (9)	Nd1—O2W	2.467 (5)
C1—O2	1.270 (9)	Nd1—O3	2.474 (5)
C1—C2	1.492 (10)	Nd1—O6^ii^	2.490 (5)
C2—N1	1.350 (10)	Nd1—O4^i^	2.508 (5)
C2—C3	1.386 (11)	Nd1—O5	2.512 (5)
C3—N2	1.328 (11)	Nd1—O1W	2.549 (6)
C3—H3	0.9300	Nd1—O2^iii^	2.557 (5)
C4—N2	1.345 (11)	Nd1—O2	2.573 (5)
C4—C5	1.379 (11)	Nd1—N1^iii^	2.765 (6)
C4—H4	0.9300	Nd1—O1	2.885 (6)
C5—N1	1.337 (10)	O2—Nd1^iii^	2.557 (5)
C5—H5	0.9300	O4—Nd1^i^	2.508 (5)
C6—O4	1.239 (9)	O6—Nd1^ii^	2.490 (5)
C6—O3	1.259 (9)	O1W—H1W	0.84 (9)
C6—C6^i^	1.553 (13)	O1W—H2W	0.85 (8)
C7—O5	1.243 (9)	O2W—H3W	0.84 (5)
C7—O6	1.247 (9)	O2W—H4W	0.84 (8)
C7—C7^ii^	1.560 (13)		
			
O1—C1—O2	123.1 (7)	O6^ii^—Nd1—O2^iii^	151.94 (19)
O1—C1—C2	120.3 (6)	O4^i^—Nd1—O2^iii^	72.00 (17)
O2—C1—C2	116.6 (6)	O5—Nd1—O2^iii^	107.89 (16)
N1—C2—C3	121.0 (7)	O1W—Nd1—O2^iii^	125.89 (18)
N1—C2—C1	115.7 (6)	O2W—Nd1—O2	76.93 (18)
C3—C2—C1	123.3 (7)	O3—Nd1—O2	133.80 (17)
N2—C3—C2	123.2 (8)	O6^ii^—Nd1—O2	113.72 (16)
N2—C3—H3	118.4	O4^i^—Nd1—O2	76.60 (17)
C2—C3—H3	118.4	O5—Nd1—O2	153.11 (18)
N2—C4—C5	122.9 (7)	O1W—Nd1—O2	74.92 (18)
N2—C4—H4	118.6	O2^iii^—Nd1—O2	60.11 (19)
C5—C4—H4	118.6	O2W—Nd1—N1^iii^	97.86 (19)
N1—C5—C4	121.6 (7)	O3—Nd1—N1^iii^	72.90 (18)
N1—C5—H5	119.2	O6^ii^—Nd1—N1^iii^	128.29 (18)
C4—C5—H5	119.2	O4^i^—Nd1—N1^iii^	68.70 (19)
O4—C6—O3	126.2 (6)	O5—Nd1—N1^iii^	65.79 (18)
O4—C6—C6^i^	117.3 (8)	O1W—Nd1—N1^iii^	131.7 (2)
O3—C6—C6^i^	116.4 (8)	O2^iii^—Nd1—N1^iii^	59.62 (17)
O5—C7—O6	127.3 (6)	O2—Nd1—N1^iii^	116.80 (17)
O5—C7—C7^ii^	116.5 (8)	O2W—Nd1—O1	66.01 (18)
O6—C7—C7^ii^	116.2 (8)	O3—Nd1—O1	128.48 (16)
C5—N1—C2	116.2 (7)	O6^ii^—Nd1—O1	66.65 (16)
C5—N1—Nd1^iii^	126.3 (5)	O4^i^—Nd1—O1	113.54 (17)
C2—N1—Nd1^iii^	114.6 (5)	O5—Nd1—O1	120.34 (17)
C3—N2—C4	114.9 (7)	O1W—Nd1—O1	64.46 (19)
O2W—Nd1—O3	149.08 (18)	O2^iii^—Nd1—O1	99.62 (15)
O2W—Nd1—O6^ii^	83.06 (18)	O2—Nd1—O1	47.38 (16)
O3—Nd1—O6^ii^	80.47 (18)	N1^iii^—Nd1—O1	158.20 (17)
O2W—Nd1—O4^i^	139.97 (17)	C1—O1—Nd1	87.4 (4)
O3—Nd1—O4^i^	65.15 (16)	C1—O2—Nd1^iii^	122.1 (4)
O6^ii^—Nd1—O4^i^	135.42 (18)	C1—O2—Nd1	101.6 (4)
O2W—Nd1—O5	76.22 (19)	Nd1^iii^—O2—Nd1	119.89 (19)
O3—Nd1—O5	73.09 (18)	C6—O3—Nd1	120.1 (4)
O6^ii^—Nd1—O5	64.36 (16)	C6—O4—Nd1^i^	119.1 (4)
O4^i^—Nd1—O5	124.93 (17)	C7—O5—Nd1	121.0 (4)
O2W—Nd1—O1W	130.01 (19)	C7—O6—Nd1^ii^	121.8 (4)
O3—Nd1—O1W	68.27 (19)	Nd1—O1W—H1W	125 (5)
O6^ii^—Nd1—O1W	71.92 (19)	Nd1—O1W—H2W	117 (6)
O4^i^—Nd1—O1W	69.52 (19)	H1W—O1W—H2W	105 (8)
O5—Nd1—O1W	125.09 (18)	Nd1—O2W—H3W	124 (5)
O2W—Nd1—O2^iii^	68.90 (17)	Nd1—O2W—H4W	128 (5)
O3—Nd1—O2^iii^	124.64 (16)	H3W—O2W—H4W	106 (8)

Symmetry codes: (i) −*x*+2, −*y*+2, −*z*+2; (ii) −*x*+1, −*y*+1, −*z*+1; (iii) −*x*+2, −*y*+1, −*z*+2.

### Hydrogen-bond geometry (Å, °)

**Table:**

*D*—H···*A*	*D*—H	H···*A*	*D*···*A*	*D*—H···*A*
O1W—H1W···O1^iv^	0.84 (9)	2.02 (6)	2.707 (8)	139 (7)
O1W—H2W···O6^v^	0.85 (8)	2.07 (4)	2.838 (8)	152 (6)
O2W—H3W···N2^vi^	0.84 (5)	2.50 (5)	3.229 (9)	145 (6)
O2W—H4W···O4^vii^	0.84 (8)	2.13 (4)	2.872 (8)	147 (7)

Symmetry codes: (iv) −*x*+2, −*y*+1, −*z*+1; (v) *x*+1, *y*, *z*; (vi) *x*−1, *y*, *z*; (vii) *x*, *y*−1, *z*.
